# Supplementary material for: A high HIV-1 strain variability in London, UK, revealed by full-genome analysis: Results from the ICONIC project
Source: PLoS One. 2018 Feb 1;13(2):e0192081. doi: 10.1371/journal.pone.0192081 (PMC5794160; doi:10.1371/journal.pone.0192081)
Supplement: S1 File — Includes Supporting Results, Figuress A, B and C, and Tables A and B. (PDF) [file pone.0192081.s001.pdf]

Supporting Information for:

A High HIV-1 Strain Variability in London, UK, Revealed by Full-Genome Analysis: Results from the ICONIC Project

Gonzalo Yebra[1,*], Dan Frampton[2], Tiziano Gallo Cassarino[2], Jade Raffle[2,3], Jonathan Hubb[4], R. Bridget Ferns[3,5], Laura Waters[6], William Tong[4], Zisis Kozlakidis[2,7], Andrew Hayward[8], Paul Kellam[9], Deenan Pillay[2], Duncan Clark[4,10], Eleni Nastouli[3,11] and Andrew J. Leigh Brown[1] on behalf of the ICONIC consortium^.

[1] Institute of Evolutionary Biology, University of Edinburgh, Edinburgh, UK
[2] UCL Division of Infection and Immunity, Faculty of Medical Sciences, London, UK
[3] Department of Clinical Virology, UCL Hospital NHS Foundation Trust, London, UK
[4] Department of Virology, Barts Health NHS Trust, London, UK
[5] NIHR UCLH/UCL Biomedical Research Centre, London, UK
[6] Department of HIV Medicine, Mortimer Market Centre, Central & North West London NHS Trust, London, UK
[7] Queen Mary University, London, UK
8 UCL Institute of Disease Informatics, Farr Institute of Health Informatics Research, London, UK
[9] UCL Institute of Epidemiology and Health Care, London, UK
[10] Division of Infectious Diseases, Department of Medicine, Imperial College London, London, UK
[11] School of Life Sciences, University of Glasgow. Glasgow, UK
[12] Department of Population, Policy and Practice, UCL GOS Institute of Child Health, London, UK

* Correspondence to GY (Gonzalo.Yebra@ed.ac.uk)

This file contents:

- Supporting Results (page 2).
- Fig A (page 5).
- Fig B (page 6).
- Fig C (page 7)
- Table A (page 9).
- Table B (page 18).

Supporting Results

Unique recombinant forms (URFs)

We provide here a detailed description for the recombination patterns of the HIV-1 Unique Recombinant Forms (URFs) not covered in the Main Text. For those URF presenting breakpoints in the PR+RT pol region, genetically similar pol sequences were searched within the UK HIV Drug Resistance database (UKHDRD).

B/C. We found 3 URF_BC, of which two formed a transmission pair (MF109542 and & MF109595, genome-wide similarity=97.1%) that involved common breakpoints –a subtype B fragment covering most of *gag-pol* in a subtype C background. The subtype B regions were genetically close to GenBank sequences sampled in the US and Germany (similarity=94%), whereas the subtype C *env* segment was close to the South African epidemics with the most similar sequences found through BLAST being sampled in Botswana (similarity=86%). The genome-wide genetic similarity between these two URF_BC was 97.12%. For the third, unrelated URF_BC found here (MF109486), the subtype C *env* fragment also presented high similarity with GenBank sequences from South Africa and Malawi (up to 87% of similarity), whereas the subtype B portion in *gag* was similar to GenBank sequences from Spain, the US and the UK (up to 94%).

B/G/CRF02_AG (MF109355). This URF was classified as A1/B/G by jpHMM, but all subtype A1 and one subtype G segments (in *gag-pol*) belonged to CRF02_AG; whereas the subtype G segments covering *vpu* and *nef* clustered with pure G reference sequences. Furthermore, many of these regions assigned to CRF02_AG and the small subtype B region in *env* seemed to be closely related to CRF56_cpx, a recombinant described among MSM subjects in France in 2013 [1]. However, the URF_02BG reported here showed different breakpoints than those found in CRF56_cpx (made up of B, G and CRF02_AG as well) in the *env* gene. The lack of *pol* sequence prevents for a further and better study of the missing subtype B segments.

B/CRF02_AG. We found 3 URF_02B. In the first one (MF109397), subtype B represented most of the genome, whereas the *env* gene corresponded CRF02_AG. The subtype B PR+RT *pol* segment presented high similarity with UK sequences from the UKHDRD (similarity=98%), whereas the closest GenBank sequences to the CRF02_AG segment were from Cameroon and France (87%). In the second case (MF109508) only part of the *gag* gene belonged to CRF02_AG with the rest of the genome corresponding to subtype B. The closest GenBank sequences to the CRF02_AG parental (similarity=96%) were sampled in Nigeria. Finally, the last URF_02B (MF109672) corresponded to subtype B in the integrase coding region (*pol*) within a CRF02_AG genomic background. The PR+RT *pol* segment presented high similarity with CRF02_AG GenBank and UKHDRD sequences from the UK (97%), which may suggest that the parental CRF02_AG variant was circulating locally.

B/F1/CRF02_AG (MF109637). The longest part of the genome corresponded to subtype F1, in which this sequence clustered very closely with two other pure subtype F1 sequences in the cohort (MF109526and MF109434) (similarity in the concatenated, 5,679bp-long F1 segments=95.4% and 95.66% respectively). This fact would point to this F1 lineage (already circulating in the UK) as the parental F1 variant, and possibly an evidence that the recombination that generated this URF_02BF1 took place in the UK. The closest GenBank sequences to these subtype F1 segments were from Brazil in both *gag* and *env* regions (similarity=92% and 88% respectively). For the subtype B *pol* region (1258bp) we could not find closely related sequences (maximum similarity within UKHDRD=94%), whereas both CRF02_AG segments (in *pol* with 292bp and in *env* with 850bp) were very close to a "pure" CRF02_AG sequence in our cohort (MF109500, similarity=98% and 94% in both segments respectively) and, to a lesser extent, to a GenBank lineage of sequences sampled predominantly in France (similarity=87%). The presence of putative parental F1 and CRF02_AG lineages within our dataset could be evidence of a recombination event occurred at least in Western Europe. The PR+RT region, which covered two breakpoints (F1/CRF02_AG/B), had 4 similar sequences (95%) in the UKHDRD, but the jpHMM analysis of them showed the same CRF02_AG/B breakpoint but no subtype F1 region. The closest GenBank PR+RT sequences were CRF02_AG from Burkina Faso, Senegal and Cameroon (93%) but corresponded to pure subtype G sequences.

B/CRF01_AE. We found 4 URF_01B in our dataset. Two of them represented a transmission pair of 2 URF_01B (MF109404and MF109516, genome-wide similarity=97.7%) which shared the same recombination breakpoints (although part of one of the sequences was missing). The sequence that covered the PR+RT *pol* region presented a CRF01_AE/B breakpoint in it, which allowed identifying 4 highly similar (>99%) *pol* sequences in the UKHDRD with the same breakpoint according to jpHMM. The most similar GenBank CRF01_AE sequences were from Thailand in *gag* (97%) and *env* (88%). The third URF_01B (MF109614) presented only 2 short portions of subtype B in *pol* and *vpu*. Despite the fact that the breakpoint in *pol* was similar to one found in the URF_01B transmission pair described above, they were not phylogenetically close. Again, we looked for similar sequences to the PR+RT region within the UKHDRD and discovered 4 similar (>96%) samples with the same recombination breakpoint. The last URF_01B (MF109462) also had two, but different, subtype B segments in integrase (*pol*) and *vpr-tat*, being unrelated to the cases above in the phylogenetic analyses and without breakpoints in PR+RT.

B/F1. We found a pair of 2 URF_BF1 (MF109665and MF109674, genome-wide similarity=96.8%) which shared identical breakpoints. They were closely related to B/F1 recombinants from South America (e.g. Bolivia, Argentina, Brazil), particularly to CRF12_BF and CRF17_BF but they presented difference recombination breakpoints. We could not find highly similar sequences already circulating in the UK that could clarify whether the recombination events that generated these URF_BF1 happened in the UK or abroad.

A1/C. We found 2 unrelated URF_A1C with different recombination patterns. The first URF_A1C (MF109562) presented a subtype A1 fragment in *gag* that was close to GenBank sequences from Kenya (similarity=94%), but the rest of the genome belonged to subtype C. The *pol* and *env* genes were similar to GenBank sequences from Mozambique and Malawi, respectively (similarity=97% and 86%). The second URF_A1C detected (MF109587) was close to GenBank subtype C sequences from South Africa in *gag* (94%) whereas its putative subtype A1 *env* gene represented a divergent lineage that actually branched outside the subtype A1/A2 crown in the ML trees. A BLAST search of this 1379bp-long segment allowed to find two URF_A1C sampled in Durban, South Africa (accession numbers AF411965 and DQ093606) that presented very similar recombination patterns and clustered together with the URF_A1C reported here after including them in the phylogenetic trees. However, they did not present a particularly high genome-wide similarity (89.7% and 90.4% respectively).

A1/C/D (MF109445). The subtypes C and D segments of this URF_A1CD were phylogenetically close to the recombinant CRF10_CD described in Tanzania in 2001 [2]. Indeed, the closest GenBank sequences to this URF were from Eastern Africa: Kenya for the subtype D *gag* (similarity=92%), the subtype A1 *pol* (95%) and the subtype C *vpu-env* (86%), and Uganda for the subtype D *env* (87%). We found 3 highly similar (identity>98%) PR+RT sequences in the UKHDRD that shared the same recombination breakpoints.

C/J (MF109649). This URF_CJ contains a small subtype J fragment roughly covering gp120 coding region (*env* gene), but most of the genome is subtype C. In the subtype J fragment, this URF clusters closely with CRF49_cpx, a recombinant between subtypes A1, C, J and K that circulates in the Gambia [3]. However, it did not share breakpoints with that CRF.

A1/G/J (MF109629). Unlike the previous case, this URF's fragments belonging to subtypes A1, G and J did not correspond to an established CRF, but to pure parental variants. The subtype G segments covered most of the genome, and was close to GenBank sequences from Nigeria/Cameroon (similarity=92% in *gag*, 95% in *pol*, 84% in *env*). The A1 segments were close to GenBank sequences from Kenya and Rwanda (similarity=93% in *gag*-protease and 92% in *env*). The subtype J portions were relatively close to CRF09_cpx, a recombinant described in West Africa but with a likely origin in Central Africa [4]. We could not find sequences that were close to the PR+RT *pol* region in either the UKHDRD or GenBank: the closest sequences had a low (<94%) similarity and were pure subtype G.

D/G (MF109700). The closest GenBank sequences to the subtype D *pol* segment (945bp long) were from Uganda and Kenya (similarity=95%). The subtype G segments presented high similarity to sequences from Uganda and Nigeria in *gag*, *pol* and *env* (similarity=92%, 94% and 86% respectively).

A1/G/CRF01 (MF109686). The jpHMM analysis provided a recombination pattern that turned out to be more complex when SimPlot and Rega analyses provided, and which was eventually confirmed by ML trees of the recombinant fragments. Namely, this URF_01AG presented extra breakpoints between CRF01_AE and subtype A1 in regions (*gag* and *vif-vpr*) initially assigned only to the latter, obscured by the similarity between these two variants. Furthermore, some segments that were assigned to subtype A1 were relatively close to CRF02_AG, although this variant was eventually ruled out as potential parental. The PR+RT *pol* region corresponded entirely to subtype G, and the closest GenBank sequences were from Central Africa (Democratic Republic of Congo and Cameroon, similarity=94%).

G/CRF02_AG. We found 2 cases of recombination between CRF02_AG and its parental strain, subtype G. Although CRF02_AG originated in the early 1970s [5], it still shares a high similarity with subtype G in some genomic regions, which complicated the detection of breakpoints. In the first URF_02G found (MF109402), most of the genome belonged to CRF02_AG, whereas two fragments of the *env* gene were ascribed to subtype G. The closest GenBank sequences to the CRF02_AG segments were sampled in Cameroon and Angola (similarity=94% in *pol*), whereas the two subtype G were similar to samples from Cameroon and Nigeria (similarity~87%). For the second URF_02G (MF109644), CRF02_AG was the parental variant for the whole *gag* region and fragments of *env*, with the rest of the sequence corresponding to subtype G, however, the *vif-vpr* region remained difficult to assign to either parental variants. The closest GenBank sequences to both CRF02_AG and G segments were from Nigeria.

References for Supporting Results

1. Leoz M, Feyertag F, Charpentier C, Delaugerre C, Wirden M, Lemee V, et al. Characterization of CRF56_cpx, a new circulating B/CRF02/G recombinant form identified in MSM in France. AIDS. 2013; 27: 2309-12.
2. Koulinska IN, Ndung'u T, Mwakagile D, Msamanga G, Kagoma C, Fawzi W, et al. A new human immunodeficiency virus type 1 circulating recombinant form from Tanzania. AIDS Res Hum Retroviruses. 2001; 17: 423-31.
3. de Silva TI, Turner R, Hué S, Trikha R, van Tienen C, Onyango C, et al. HIV-1 subtype distribution in the Gambia and the significant presence of CRF49_cpx, a novel circulating recombinant form. Retrovirology. 2010; 7: 82.
4. Delatorre E, Bello G. Time-scale of minor HIV-1 complex circulating recombinant forms from Central and West Africa. BMC Evol Biol. 2016; 16: 249.
5. Faria NR, Suchard MA, Abecasis A, Sousa JD, Ndembi N, Bonfim I, et al. Phylodynamics of the HIV-1 CRF02_AG clade in Cameroon. Infect Genet Evol. 2012; 12: 453-60.

Fig A. Alignment of the 375 consensus HIV-1 ICONIC genome sequences. The diagram shows the HIV-1 genome map and HXB2 coordinates for the limits of the main genes. The arrows indicate the position of the primer sets used for amplification (see Methods section of the main text).

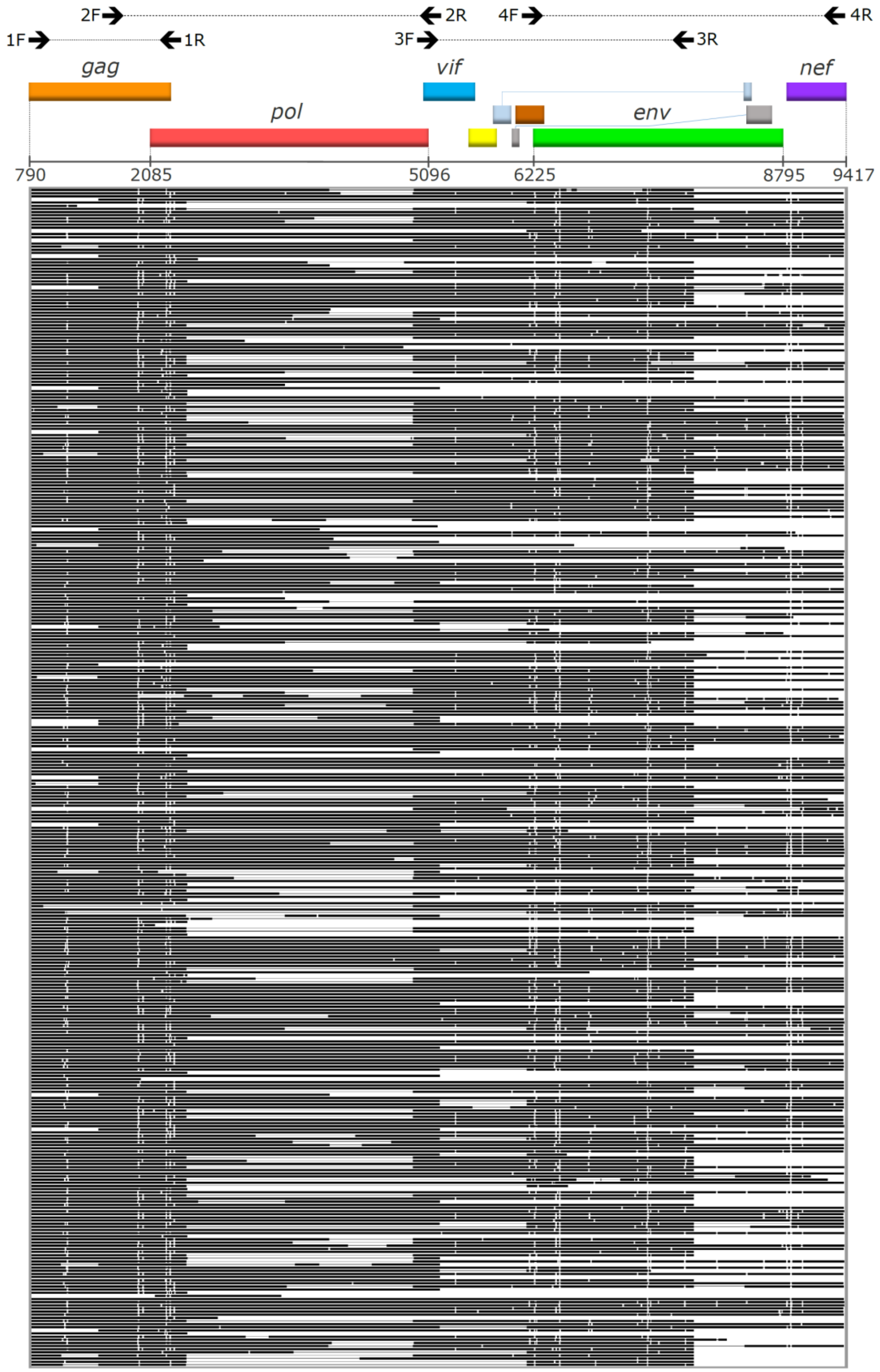

Fig B. Scatterplot showing the correlation between HIV-1 viral load and sequence length. The regression line (R^2 = 0.05; p = 0.0026) is shown in blue.

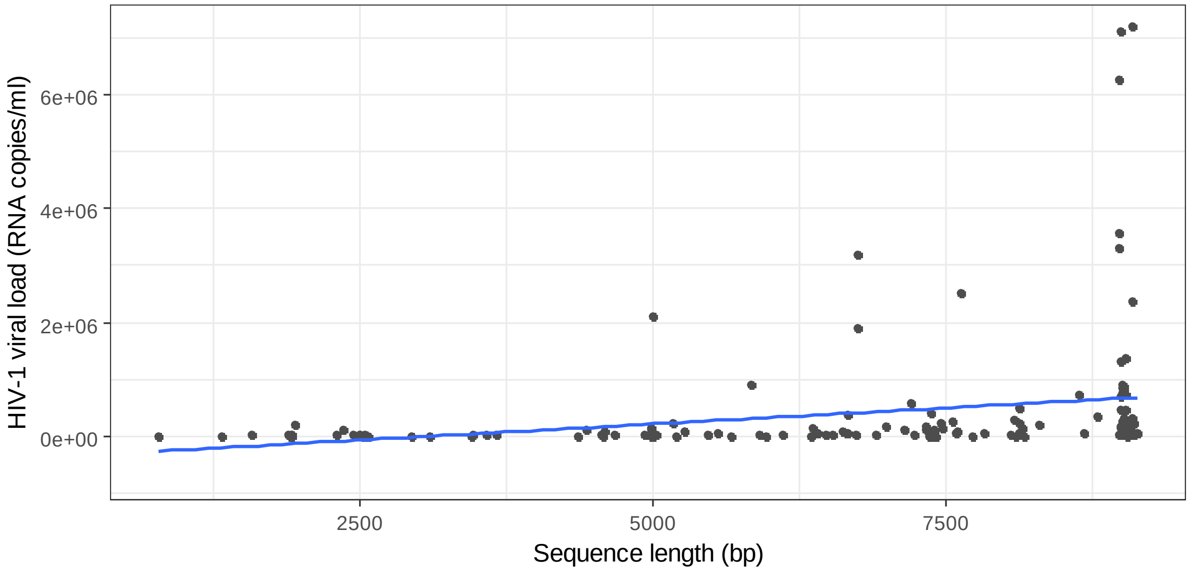

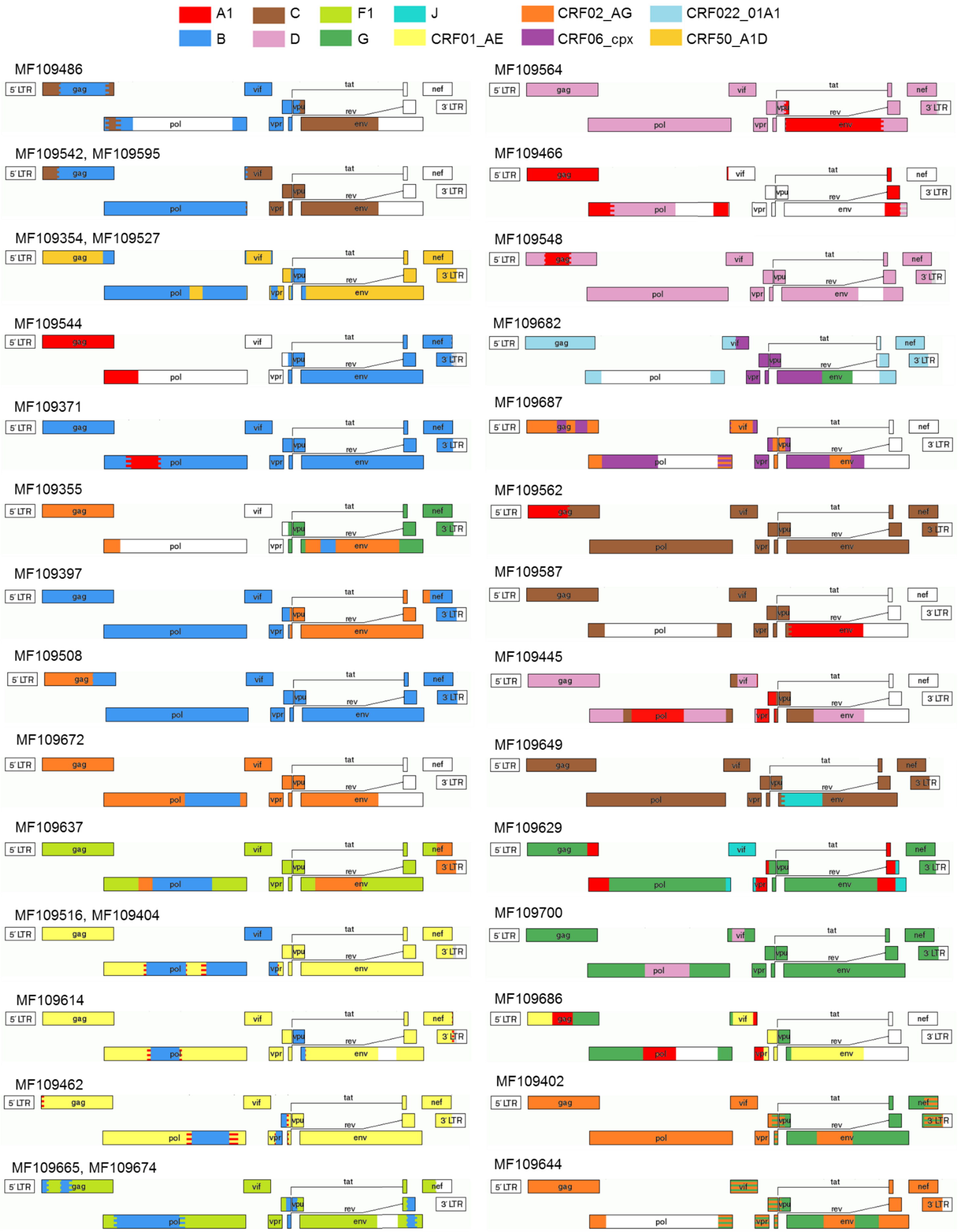
A1
C
F1
J
CRF02_AG
CRF022_01A1
B
D
G
CRF01_AE
CRF06_cpx
CRF50_A1D
MF109486
MF109564
MF109542, MF109595
MF109466
MF109354, MF109527
MF109548
MF109544
MF109682
MF109371
MF109687
MF109355
MF109562
MF109397
MF109587
MF109508
MF109445
MF109672
MF109649
MF109637
MF109629
MF109516, MF109404
MF109700
MF109614
MF109686
MF109462
MF109402
MF109665, MF109674
MF109644
5' LTR
gag
pol
vif
vpr
vpu
tat
rev
env
nef
3' LTR

Fig C (previous page). Recombination patterns of the 32 unique recombinant forms found in the ICONIC dataset. The colours denote the parental variants involved in the recombination. White colour corresponds to sequence gaps. Note that for URFs pairs that presented the same recombination breakpoints, only one diagram is shown.

Table A. Genbank accession number and metadata for the 375 assembled HIV-1 genomes used in this study.

	Accession no.	Isolation date	HIV-1 subtype	Length (bp)
**1**	MF109362	19/11/2013	B	5668
**2**	MF109352	21/11/2013	G	8298
**3**	MF109353	22/11/2013	B	7344
**4**	MF109354	21/11/2013	URF (B/CRF50_A1D)	8085
**5**	MF109355	21/11/2013	URF (B/G/CRF02_AG)	5489
**6**	MF109356	14/11/2013	C	1066
**7**	MF109357	20/11/2013	CRF06_cpx	5288
**8**	MF109358	20/11/2013	CRF01_AE	8978
**9**	MF109359	22/11/2013	B	9041
**10**	MF109360	22/11/2013	F1	8038
**11**	MF109361	22/11/2013	A1	7416
**12**	MF109363	22/11/2013	B	9044
**13**	MF109364	26/11/2013	B	9054
**14**	MF109365	26/11/2013	CRF19_cpx	1262
**15**	MF109366	26/11/2013	C	8998
**16**	MF109367	26/11/2013	C	9049
**17**	MF109368	26/11/2013	B	3089
**18**	MF109369	26/11/2013	B	9008
**19**	MF109370	27/11/2013	CRF01_AE	8263
**20**	MF109371	27/11/2013	URF (A1/B)	9011
**21**	MF109372	27/11/2013	B	9038
**22**	MF109383	19/11/2013	CRF06_cpx	8064
**23**	MF109373	25/11/2013	B	2560
**24**	MF109374	04/12/2013	CRF02_AG	6348
**25**	MF109375	05/12/2013	B	3450
**26**	MF109376	04/12/2013	B	9045
**27**	MF109377	06/12/2013	CRF01_AE	6757
**28**	MF109378	04/12/2013	CRF18_cpx	8944
**29**	MF109379	04/12/2013	C	7320
**30**	MF109380	06/12/2013	CRF02_AG	1923
**31**	MF109381	06/12/2013	CRF02_AG	9052
**32**	MF109382	02/12/2013	G	7234
**33**	MF109388	19/11/2013	B	9089
**34**	MF109384	07/12/2013	CRF02_AG	8546
**35**	MF109385	02/12/2013	B	7357
**36**	MF109386	16/12/2013	B	7425
**37**	MF109387	11/12/2013	C	7357
**38**	MF109389	19/11/2013	C	9022
**39**	MF109390	22/11/2013	B	3825
**40**	MF109391	21/11/2013	A1	8162
**41**	MF109392	21/11/2013	CRF01_AE	7788

	Accession no.	Isolation date	HIV-1 subtype	Length (bp)
**42**	MF109393	21/11/2013	B	4555
**43**	MF109402	03/01/2014	URF (G/CRF02_AG)	9095
**44**	MF109394	14/01/2014	C	6423
**45**	MF109395	23/01/2014	B	9038
**46**	MF109396	22/01/2014	B	9048
**47**	MF109397	03/01/2014	URF (B/CRF02_AG)	9030
**48**	MF109398	22/01/2014	B	4962
**49**	MF109399	15/01/2014	C	2517
**50**	MF109400	23/01/2014	B	9000
**51**	MF109401	22/01/2014	B	4356
**52**	MF109413	03/01/2014	C	9013
**53**	MF109403	15/01/2014	C	7364
**54**	MF109404	23/01/2014	URF (B/CRF01_AE)	4980
**55**	MF109405	25/01/2014	G	4970
**56**	MF109406	24/01/2014	CRF02_AG	4417
**57**	MF109407	24/01/2014	CRF01_AE	9051
**58**	MF109408	24/01/2014	B	9007
**59**	MF109409	20/01/2014	CRF02_AG	5957
**60**	MF109410	24/01/2014	C	1891
**61**	MF109411	25/01/2014	B	7341
**62**	MF109412	22/01/2014	C	9079
**63**	MF109424	23/12/2013	A1	2929
**64**	MF109414	22/01/2014	C	3580
**65**	MF109415	20/01/2014	C	1901
**66**	MF109416	28/01/2014	B	1907
**67**	MF109417	28/01/2014	CRF13_cpx	9099
**68**	MF109418	28/01/2014	B	9066
**69**	MF109419	20/01/2014	B	4973
**70**	MF109420	29/01/2014	B	8289
**71**	MF109421	29/01/2014	CRF02_AG	5031
**72**	MF109422	29/01/2014	B	9083
**73**	MF109423	29/01/2014	B	6658
**74**	MF109430	07/01/2014	B	6905
**75**	MF109425	31/01/2014	C	7731
**76**	MF109426	29/01/2014	B	8991
**77**	MF109427	29/01/2014	A1	9045
**78**	MF109428	29/01/2014	B	8007
**79**	MF109429	29/01/2014	C	5790
**80**	MF109431	14/01/2014	CRF02_AG	6091
**81**	MF109432	14/01/2014	CRF02_AG	8923
**82**	MF109433	13/01/2014	B	6629
**83**	MF109434	29/01/2014	F1	8988
**84**	MF109435	29/01/2014	C	9074

	Accession no.	Isolation date	HIV-1 subtype	Length (bp)
**85**	MF109436	29/01/2014	CRF02_AG	6749
**86**	MF109437	29/01/2014	C	9051
**87**	MF109438	29/01/2014	F1	5200
**88**	MF109439	29/01/2014	CRF02_AG	8995
**89**	MF109440	01/02/2014	B	9056
**90**	MF109441	01/02/2014	C	9020
**91**	MF109442	26/11/2013	C	4978
**92**	MF109443	23/01/2014	C	2175
**93**	MF109444	06/02/2014	B	7264
**94**	MF109445	04/02/2014	URF (A1/C/D)	7327
**95**	MF109446	05/02/2014	B	9013
**96**	MF109447	18/01/2014	B	9066
**97**	MF109448	05/02/2014	CRF02_AG	7408
**98**	MF109449	03/02/2014	B	8989
**99**	MF109450	07/02/2014	B	7349
**100**	MF109451	13/02/2014	B	6649
**101**	MF109452	12/02/2014	B	8987
**102**	MF109453	13/02/2014	B	9043
**103**	MF109454	14/02/2013	B	9009
**104**	MF109455	14/02/2014	B	7343
**105**	MF109456	11/02/2014	B	9024
**106**	MF109457	22/01/2014	C	5513
**107**	MF109458	14/02/2014	CRF01_AE	1923
**108**	MF109459	14/02/2014	B	4607
**109**	MF109460	14/02/2014	B	2513
**110**	MF109461	14/02/2014	B	8392
**111**	MF109462	13/02/2014	URF (B/CRF01_AE)	9007
**112**	MF109463	14/02/2014	CRF49_cpx	3709
**113**	MF109464	14/02/2014	B	4885
**114**	MF109465	18/02/2014	B	5322
**115**	MF109466	28/02/2014	URF (A1/D)	4164
**116**	MF109467	28/02/2014	C	7120
**117**	MF109468	28/02/2014	B	8315
**118**	MF109469	26/02/2014	B	8491
**119**	MF109470	27/02/2014	C	2087
**120**	MF109471	11/12/2013	CRF01_AE	9064
**121**	MF109472	12/12/2013	B	9057
**122**	MF109473	12/12/2013	B	7332
**123**	MF109474	13/12/2013	CRF06_cpx	9023
**124**	MF109475	11/12/2013	B	8992
**125**	MF109476	11/12/2013	CRF03_AB	9058
**126**	MF109477	13/12/2013	C	3871
**127**	MF109478	28/11/2013	B	7419

	Accession no.	Isolation date	HIV-1 subtype	Length (bp)
**128**	MF109479	05/12/2013	B	8994
**129**	MF109480	13/12/2013	B	9088
**130**	MF109481	12/12/2013	B	1907
**131**	MF109482	13/12/2013	CRF02_AG	2933
**132**	MF109483	14/12/2013	B	8089
**133**	MF109484	12/12/2013	D	1911
**134**	MF109485	17/12/2013	B	8745
**135**	MF109486	16/12/2013	URF (B/C)	5157
**136**	MF109487	17/12/2013	B	9010
**137**	MF109488	16/12/2013	B	7828
**138**	MF109489	17/12/2013	F1	5460
**139**	MF109490	20/12/2013	B	8173
**140**	MF109491	10/12/2013	C	1566
**141**	MF109492	18/12/2013	B	5000
**142**	MF109493	18/12/2013	C	7771
**143**	MF109494	18/12/2013	B	9099
**144**	MF109495	18/12/2013	B	7353
**145**	MF109496	19/12/2013	CRF01_AE	4568
**146**	MF109497	18/12/2013	C	1899
**147**	MF109498	17/12/2013	B	1918
**148**	MF109499	18/12/2013	B	8926
**149**	MF109500	18/12/2013	CRF02_AG	7583
**150**	MF109501	18/12/2013	B	8984
**151**	MF109502	20/12/2013	A1	7374
**152**	MF109503	18/12/2013	C	1075
**153**	MF109504	31/12/2013	C	9067
**154**	MF109505	31/12/2013	CRF01_AE	6310
**155**	MF109506	30/12/2013	B	8987
**156**	MF109507	05/11/2013	C	6742
**157**	MF109508	31/12/2013	URF (B/CRF02_AG)	9023
**158**	MF109509	31/12/2013	B	7332
**159**	MF109510	29/12/2013	C	7353
**160**	MF109518	01/05/2014	B	6665
**161**	MF109511	02/05/2014	CRF02_AG	5999
**162**	MF109512	02/05/2014	CRF14_BG	6064
**163**	MF109513	01/05/2014	C	8865
**164**	MF109514	24/04/2014	C	8491
**165**	MF109515	08/05/2014	CRF02_AG	7949
**166**	MF109516	08/05/2014	URF (B/CRF01_AE)	9024
**167**	MF109517	07/05/2014	C	5246
**168**	MF109527	25/04/2014	URF (B/CRF50_AD)	8994
**169**	MF109519	06/05/2014	CRF02_AG	3461
**170**	MF109520	21/05/2014	A1	3818

	**Accession no.**	**Isolation date**	**HIV-1 subtype**	**Length (bp)**
**171**	MF109521	27/05/2014	CRF01_AE	3888
**172**	MF109522	29/05/2014	C	9097
**173**	MF109523	28/05/2014	B	9012
**174**	MF109524	07/05/2014	B	9039
**175**	MF109525	08/05/2014	B	8877
**176**	MF109526	28/05/2014	F1	9029
**177**	MF109532	24/04/2014	D	9031
**178**	MF109528	29/05/2014	C	7294
**179**	MF109529	03/06/2014	A1	3061
**180**	MF109530	27/05/2014	A1	4552
**181**	MF109531	19/05/2014	B	1919
**182**	MF109533	25/04/2014	B	9058
**183**	MF109534	24/04/2014	B	9077
**184**	MF109535	02/05/2014	C	9023
**185**	MF109536	30/04/2014	B	9013
**186**	MF109537	15/04/2014	B	1919
**187**	MF109538	10/06/2014	CRF25_cpx	9119
**188**	MF109539	09/06/2014	C	8014
**189**	MF109540	10/06/2014	B	8989
**190**	MF109541	06/06/2014	C	1244
**191**	MF109542	04/06/2014	URF (B/C)	7394
**192**	MF109543	02/06/2014	B	9040
**193**	MF109544	02/06/2014	URF (A1/B)	5826
**194**	MF109545	18/06/2014	B	4970
**195**	MF109546	16/06/2014	B	8729
**196**	MF109547	18/06/2014	CRF02_AG	9014
**197**	MF109548	19/06/2014	URF (A1/D)	8494
**198**	MF109549	20/06/2014	C	1648
**199**	MF109550	19/06/2014	CRF02_AG	9058
**200**	MF109551	19/06/2014	B	9100
**201**	MF109552	19/06/2014	B	7345
**202**	MF109553	18/06/2014	CRF06_cpx	7337
**203**	MF109554	13/06/2014	B	3574
**204**	MF109555	10/06/2014	CRF07_BC	8979
**205**	MF109556	16/06/2014	CRF02_AG	2892
**206**	MF109557	17/06/2014	C	9035
**207**	MF109558	05/06/2014	B	9001
**208**	MF109559	17/06/2014	CRF02_AG	9047
**209**	MF109560	06/06/2014	C	8216
**210**	MF109561	12/06/2014	B	8993
**211**	MF109562	14/06/2014	URF (A1/C)	8999
**212**	MF109563	16/06/2014	C	9042
**213**	MF109564	12/06/2014	URF (A1/D)	9030

	Accession no.	Isolation date	HIV-1 subtype	Length (bp)
**214**	MF109565	11/06/2014	B	7138
**215**	MF109566	11/06/2014	B	7350
**216**	MF109567	11/06/2014	C	8101
**217**	MF109568	13/06/2014	G	9052
**218**	MF109569	12/06/2014	B	1631
**219**	MF109570	11/06/2014	B	4957
**220**	MF109571	11/06/2014	B	7067
**221**	MF109572	13/06/2014	CRF02_AG	9025
**222**	MF109580	19/02/2014	C	1893
**223**	MF109573	12/03/2014	B	7905
**224**	MF109574	27/02/2014	B	4513
**225**	MF109575	14/03/2014	CRF01_AE	7656
**226**	MF109576	14/03/2014	B	4580
**227**	MF109577	14/03/2014	B	1939
**228**	MF109578	12/03/2014	B	9015
**229**	MF109579	27/02/2014	C	3181
**230**	MF109590	14/02/2014	B	8941
**231**	MF109581	27/03/2014	C	3950
**232**	MF109582	27/03/2014	B	4702
**233**	MF109583	26/03/2014	C	5102
**234**	MF109584	19/03/2014	G	1936
**235**	MF109585	27/03/2014	CRF06_cpx	1608
**236**	MF109586	27/03/2014	C	5034
**237**	MF109587	29/03/2014	URF (A1/C)	4660
**238**	MF109588	29/03/2014	B	1929
**239**	MF109589	31/03/2014	C	8867
**240**	MF109591	19/03/2014	C	9010
**241**	MF109592	12/03/2014	C	9051
**242**	MF109593	31/10/2013	C	8987
**243**	MF109594	20/03/2014	B	8115
**244**	MF109595	20/03/2014	URF (B/C)	9053
**245**	MF109596	20/03/2014	B	9057
**246**	MF109597	20/03/2014	B	7339
**247**	MF109598	20/03/2014	G	9034
**248**	MF109599	19/03/2014	C	9039
**249**	MF109600	28/02/2014	B	5014
**250**	MF109601	26/02/2014	D	7225
**251**	MF109602	05/03/2014	B	1900
**252**	MF109603	12/03/2014	B	7267
**253**	MF109604	12/03/2014	C	6655
**254**	MF109605	20/03/2014	B	9124
**255**	MF109606	18/03/2014	C	9080
**256**	MF109607	20/03/2014	C	9010

	Accession no.	Isolation date	HIV-1 subtype	Length (bp)
**257**	MF109608	22/03/2014	B	7567
**258**	MF109609	01/04/2014	C	7391
**259**	MF109610	27/03/2014	CRF06_cpx	7361
**260**	MF109611	01/04/2014	B	4589
**261**	MF109612	02/04/2014	C	9005
**262**	MF109613	02/04/2014	A1	9093
**263**	MF109614	03/04/2014	URF (B/CRF01_AE)	8648
**264**	MF109615	03/04/2014	B	6179
**265**	MF109616	02/04/2014	B	8989
**266**	MF109617	27/03/2014	C	9042
**267**	MF109618	08/04/2014	CRF01_AE	8990
**268**	MF109619	07/04/2014	A1	3910
**269**	MF109620	07/04/2014	B	9047
**270**	MF109621	07/04/2014	CRF01_AE	9015
**271**	MF109622	07/04/2014	CRF06_cpx	1922
**272**	MF109623	02/04/2014	A1	9072
**273**	MF109624	08/04/2014	B	9068
**274**	MF109625	09/04/2014	B	4584
**275**	MF109626	09/04/2014	C	7407
**276**	MF109627	11/04/2014	B	8970
**277**	MF109628	10/04/2014	G	7369
**278**	MF109629	10/04/2014	URF (A1/G/J)	9167
**279**	MF109630	03/04/2014	B	9040
**280**	MF109631	17/03/2014	B	8556
**281**	MF109632	31/01/2014	A1	8165
**282**	MF109633	13/02/2014	B	1930
**283**	MF109634	02/04/2014	B	4581
**284**	MF109635	11/04/2014	B	1435
**285**	MF109636	10/04/2014	B	8994
**286**	MF109637	14/04/2014	URF (B/F1/CRF02_AG)	9024
**287**	MF109638	01/05/2014	B	8506
**288**	MF109639	30/04/2014	B	4986
**289**	MF109650	06/11/2013	B	2443
**290**	MF109640	28/10/2013	F1	9015
**291**	MF109641	29/10/2013	CRF02_AG	8117
**292**	MF109642	28/10/2013	B	8120
**293**	MF109643	30/10/2013	B	8640
**294**	MF109644	30/10/2013	URF (G/CRF02_AG)	6640
**295**	MF109645	29/10/2013	A1	7358
**296**	MF109646	30/10/2013	G	6732
**297**	MF109647	30/10/2013	G	9130
**298**	MF109648	25/10/2013	CRF43_02G	9085
**299**	MF109649	30/10/2013	URF (C/J)	9038

	Accession no.	Isolation date	HIV-1 subtype	Length (bp)
**300**	MF109661	11/11/2013	F1	3577
**301**	MF109651	31/10/2013	CRF06_cpx	9088
**302**	MF109652	21/10/2013	CRF02_AG	6532
**303**	MF109653	01/11/2013	C	8049
**304**	MF109654	01/11/2013	CRF09_cpx	6403
**305**	MF109655	04/11/2013	B	8679
**306**	MF109656	04/11/2013	B	9050
**307**	MF109657	04/11/2013	CRF02_AG	9033
**308**	MF109658	15/10/2013	D	4989
**309**	MF109659	31/10/2013	B	4985
**310**	MF109660	31/10/2013	C	5547
**311**	MF109672	11/11/2013	URF (B/CRF02_AG)	7403
**312**	MF109662	05/11/2013	C	6660
**313**	MF109663	05/11/2013	B	7331
**314**	MF109664	07/11/2013	B	9103
**315**	MF109665	07/11/2013	URF (B/F1)	8153
**316**	MF109666	07/11/2013	B	4665
**317**	MF109667	25/10/2013	B	9016
**318**	MF109668	07/11/2013	B	2348
**319**	MF109669	06/11/2013	C	1906
**320**	MF109670	07/11/2013	C	9025
**321**	MF109671	08/11/2013	URF (G/CRF01_AE/CRF06_cpx)	5032
**322**	MF109678	19/11/2013	CRF01_AE	7357
**323**	MF109673	23/11/2013	C	3654
**324**	MF109674	11/11/2013	URF (B/F1)	7455
**325**	MF109675	01/11/2013	B	9052
**326**	MF109676	13/11/2013	C	9016
**327**	MF109677	12/11/2013	A1	8982
**328**	MF109679	24/10/2013	A1	8982
**329**	MF109680	25/10/2013	B	9001
**330**	MF109681	25/10/2013	C	7149
**331**	MF109682	24/10/2013	URF (06_cpx/22_01A1)	4926
**332**	MF109683	13/11/2013	B	7349
**333**	MF109684	13/11/2013	C	8993
**334**	MF109685	13/11/2013	B	1934
**335**	MF109686	13/11/2013	URF (A1/G/CRF01_AE)	6477
**336**	MF109687	05/11/2013	URF (02_AG/CRF06_cpx)	6102
**337**	MF109688	04/11/2013	CRF02_AG	8787
**338**	MF109689	12/11/2013	D	8995
**339**	MF109690	15/11/2013	CRF02_AG	7377
**340**	MF109691	15/11/2013	C	4993
**341**	MF109692	13/11/2013	A1	2291
**342**	MF109693	04/09/2013	C	6616

	Accession no.	Isolation date	HIV-1 subtype	Length (bp)
**343**	MF109694	05/09/2013	B	2501
**344**	MF109695	19/11/2013	C	9046
**345**	MF109696	20/11/2013	C	5831
**346**	MF109697	22/11/2013	A1	9013
**347**	MF109698	22/11/2013	B	4578
**348**	MF109699	04/12/2013	CRF01_AE	7472
**349**	MF109700	22/11/2013	URF (D/G)	9076
**350**	MF109701	18/11/2013	CRF02_AG	7206
**351**	MF109702	20/11/2013	CRF02_AG	4558
**352**	MF109703	18/11/2013	CRF02_AG	1884
**353**	MF109704	04/10/2013	G	1315
**354**	MF109705	18/11/2013	B	9076
**355**	MF109706	22/11/2013	C	9040
**356**	MF109707	21/11/2013	C	8998
**357**	MF109708	23/11/2013	CRF02_AG	8972
**358**	MF109709	13/11/2013	CRF02_AG	9051
**359**	MF109710	10/10/2013	D	8973
**360**	MF109711	29/11/2013	B	2537
**361**	MF109712	22/11/2013	C	5171
**362**	MF109713	20/11/2013	D	9083
**363**	MF109714	16/10/2013	B	8130
**364**	MF109715	19/11/2013	C	6366
**365**	MF109716	21/11/2013	B	2493
**366**	MF109717	21/11/2013	A1	7458
**367**	MF109718	26/11/2013	CRF60_BC	7622
**368**	MF109719	26/11/2013	C	6980
**369**	MF109720	29/11/2013	B	7555
**370**	MF109721	31/10/2013	CRF02_AG	7597
**371**	MF109722	05/12/2013	B	5273
**372**	MF109723	22/11/2013	B	4982
**373**	MF109724	11/12/2013	B	5909
**374**	MF109725	07/06/2012	B	4434
**375**	MF109726	12/12/2013	B	3458

Table B. Characteristics of the 17 HIV-1 phylogenetic clusters that were consistently found across the *gag*, *pol* and *env* gene trees.

**Cluster no.**	**HIV-1 subtype**	**No. of sequences**	**Patristic genetic distance (%)**				**Boostrap support (%)**			
			**Genome**	***gag***	***pol***	***env***	**Genome**	***gag***	***pol***	***env***
**1**	B	2	2.74	2.00	1.46	4.11	100	98	100	100
**2**	B	2	0.60	1.13	0.53	0.53	100	100	100	100
**3**	B	2	4.02	2.93	2.13	6.41	100	100	98	100
**4**	B	2	0.61	0.53	0.40	0.95	100	99	100	100
**5**	B	2	2.91	3.59	2.23	3.78	100	100	99	100
**6**	C	2	1.01	0.86	0.37	1.40	100	100	100	100
**7**	C	2	2.82	1.93	1.86	4.97	100	100	100	100
**8**	C	2	0.16	0.07	0.17	0.45	100	100	100	100
**9**	01_AE	2	0.72	0.33	0.33	0.78	100	100	100	100
**10**	02_AG	2	0.08	0.00	0.03	0.21	100	100	100	100
**11**	02_AG	3	0.11	0.07	0.10	0.12	100	100	99	100
**12**	02_AG	3	4.06	3.06	2.86	5.55	100	100	100	100
**13**	06_cpx	3	5.27	5.19	2.96	8.92	100	100	100	100
**14**	URF_01B	2	1.10	1.33	0.20	2.06	100	100	100	100
**15**	URF_50B	2	3.35	1.53	2.53	5.47	100	100	100	100
**16**	URF_BC	2	2.34	2.93	2.13	3.04	100	100	100	100
**17**	URF_BF1	2	3.11	2.33	2.36	3.16	100	100	100	100
